# Tuberculous tenosynovitis of the flexor tendons of the wrist: a case report

**DOI:** 10.1186/s13104-018-3343-4

**Published:** 2018-04-10

**Authors:** Paa Kwesi Baidoo, Daniel Baddoo, Agbeko Ocloo, Daniel Agbley, Samuel Lartey, Nyonuku Akosua Baddoo

**Affiliations:** 1 Department of Surgery, Orthopedic Unit, Korle Bu Teaching Hospital, P.O. Box 77, Accra, Ghana; 2 Department of Surgery, Korle Bu Teaching Hospital, P.O. Box 77, Accra, Ghana; 3 Department of Chest Diseases (Internal Medicine), Korle Bu Teaching Hospital, P.O. Box 77, Accra, Ghana

**Keywords:** Compound palmar ganglion, Rice bodies, Melon bodies, And flexor tendon sheath

## Abstract

**Background:**

Tuberculous tenosynovitis poses a significant public health challenge, especially in developing countries. It usually affects the flexor tendons of the wrist.

**Case presentation:**

We present a case of a 65-year-old Ghanaian female. She presented a progressively enlarging mass over the volar aspect of the right wrist and palm. She did not have a previous history of tuberculosis. However, her erythrocyte sedimentation rate was high and Mantoux (purified protein derivative) test was strongly positive (more than 15 mm). Radiograph of ulna, radius, and wrist showed osteopenic changes around the distal radius. Excision biopsy of the mass was done and samples sent for histopathology comment. The findings were an inflamed, thickened synovia with rice bodies: suggestive of tuberculous tenosynovitis. Anti-tuberculous chemotherapy was commenced on the second postoperative day.

**Conclusion:**

Tuberculous tenosynovitis of the wrist is uncommon. However, in developing countries like Ghana where tuberculosis is prevalent, it should be part of the differential diagnosis of compound palmar ganglion in order to prevent delayed diagnosis and treatment.

## Background

Chronic flexor tenosynovitis of the wrist and palm (compound palmar ganglion) is usually tuberculous in origin: though other conditions like rheumatoid arthritis and systemic lupus erythematosus could cause it. It is common in males and usually involves the right hand [1]. Some cases have been reported in countries such as India, but not from Ghana as the condition is uncommon here. It may result from direct inoculation or hematogenous spread from a primary source like lungs, spine, and lymph nodes [1].

The flexor tendons of the wrist is a rare presentation of tuberculous infection [2]. Once established, it leads to chronic inflammation of all the tendons sheaths around the hand and wrist and may result in median nerve compression [3].

Diagnosis is usually delayed [4] and when this happens, it may be associated with the destruction of the underlying bone at the time the patient present to the clinic. It is therefore important to diagnose the condition early, followed by thorough surgical excision of all the tissues affected and the institution of appropriate anti-tuberculous chemotherapy [2]. The aim of this case report was to highlight the occurrence of tuberculous tenosynovitis in our environment in which it is deemed rare.

## Case presentation

A 65-year-old well-nourished Ghanaian female presented to the orthopaedic clinic of our hospital with a 2-year history of a progressively enlarging mass over the volar aspect of the right wrist and palm. It was associated with pain around the wrist: which initially responded to non-steroidal anti-inflammatory drugs until a month before she presented to the hospital. She also had stiffness at the wrist joint and paraesthesia involving the distribution of the median nerve (radial three and half fingers). These symptoms occasionally woke her up at night.

There was no history of a traumatic injury to the wrist, no contact with a patient with tuberculosis, and no symptoms suggestive of rheumatoid arthritis. The patient is a known diabetic of 10 years, which is well controlled by diet alone. She was HIV negative and has not had any vaccination against tuberculosis for the past 25 years as far as she could remember.

**Fig. 1:**
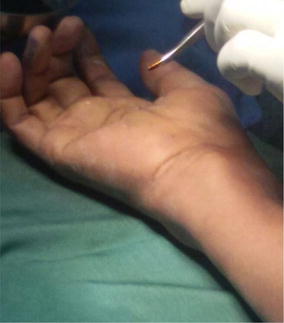
Clinical picture showing compound palmar ganglion

Examination revealed an 8 cm × 4 cm swelling on the volar aspect of the right wrist, doughy in consistency, compressible and extended about 5 cm from the wrist creases proximally, across the flexor retinaculum of the wrist into the mid-palm distally (Fig. 1). There was cross fluctuancy, wasting of the thenar muscles with associated numbness along the distribution of the median nerve. The superficial lymph nodes were not palpable. Examination of the chest and spine were essentially normal.

**Fig. 2:**
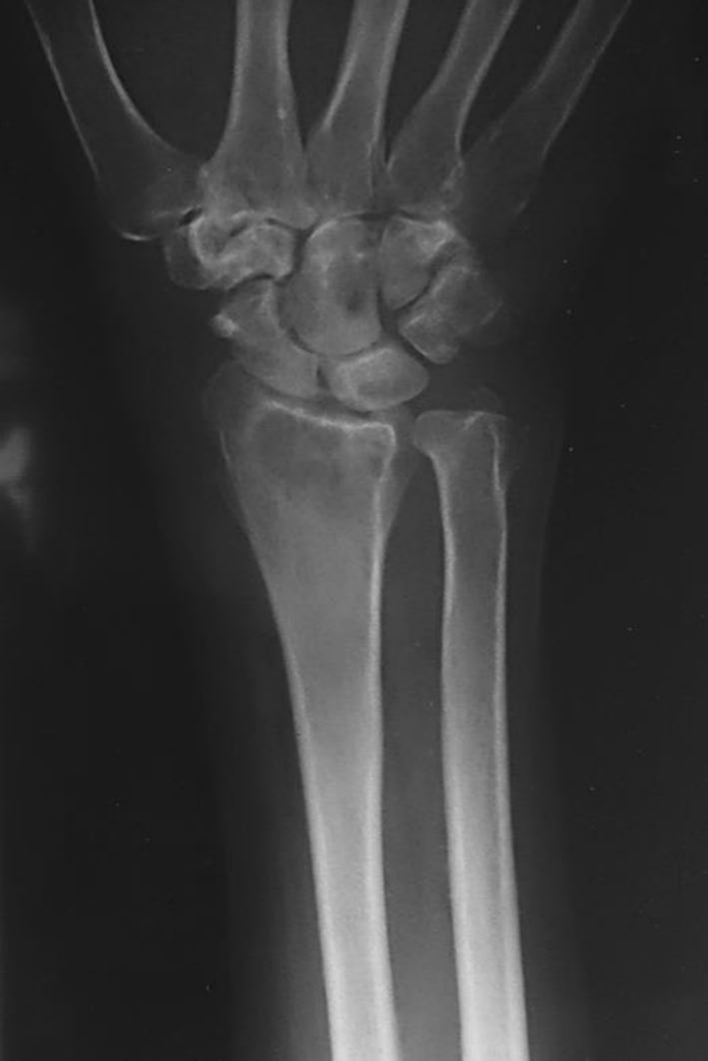
X-ray of the distal radius/ulna with the wrist showing localized osteopenia around the radiocarpal joint

X-ray of the wrist showed localized osteopenia around the radio-carpal joint (Fig. 2). Chest X-ray was normal. Erythrocyte sedimentation rate (ESR) was raised (94 mm fall/h) and Mantoux (purified protein derivative) test was strongly positive (more than 15 mm). However, examination of the sputum did not yield any tuberculous bacilli.

**Fig. 3:**
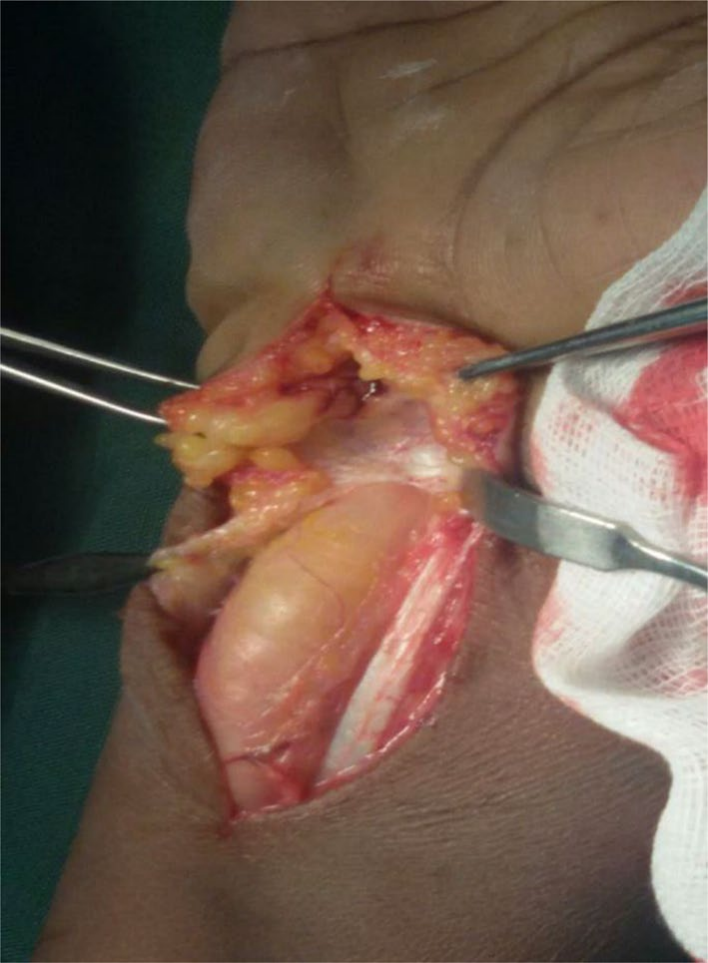
Single fluctuant mass

**Fig. 4:**
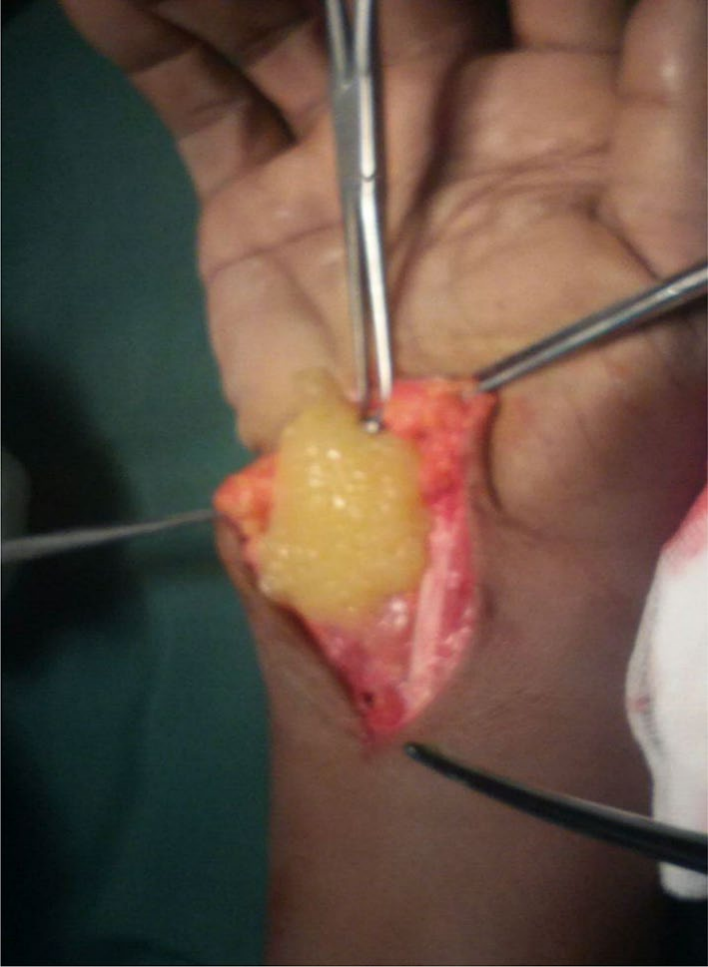
Clinical picture showing rice bodies

**Fig. 5:**
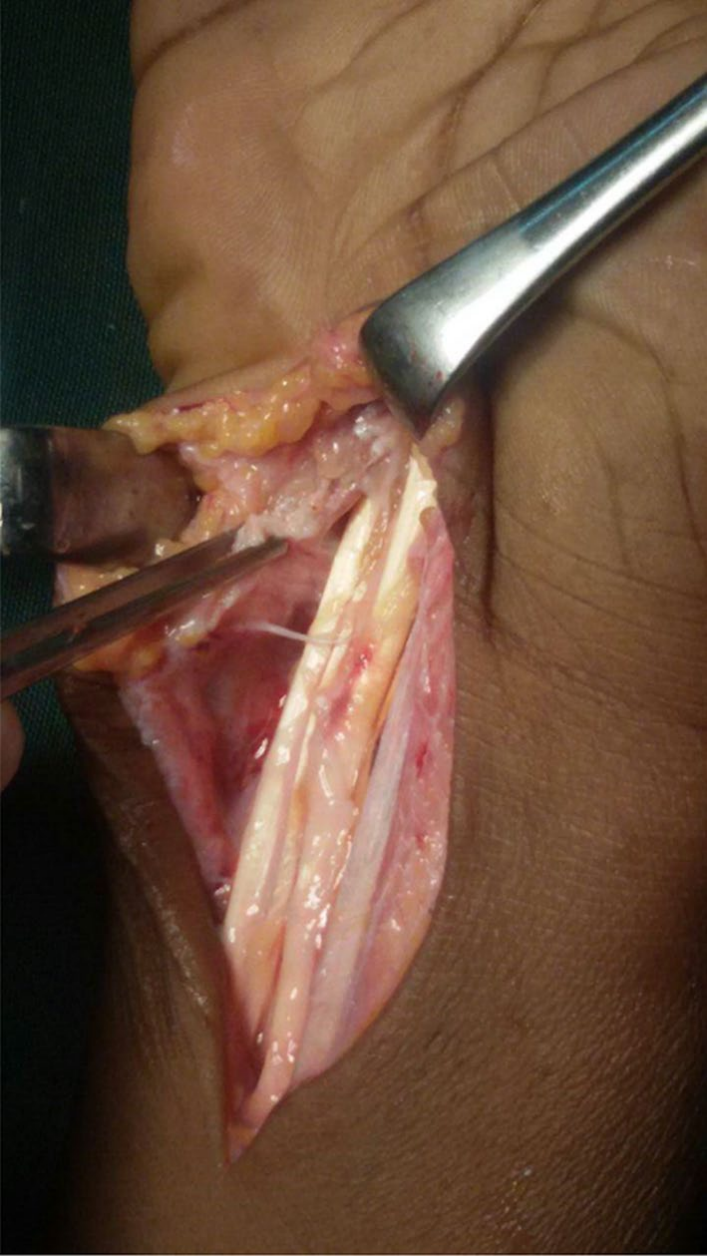
Total synovectomy done

At surgery, the mass extended across the flexor retinaculum (Fig. 3) and contained yellowish fluid with multiple rice bodies. The flexor tendons sheaths were also thickened (Fig. 4). The median nerve was found to be pale: flattened in its course through the carpal tunnel and was dumbbell shaped in appearance. Total tenosynovectomy was done (Fig. 5) and the forearm subsequently placed in a below elbow volar plaster slab for 2 weeks. The specimen sent for histopathology, culture, and sensitivity. Histopathology showed specimen with granulomatous lesions with Langerhans giant cells. Culture was positive for acid-fast bacilli. Anti-tuberculous chemotherapy was started on the second postoperative day on account of the clinical findings and appearance of the specimen at surgery. The antituberculous chemotherapy comprised of isoniazid, ethambutol, pyrazinamide, and rifampicin for the first 3 months and isoniazid and rifampicin for another 6 months.

**Fig. 6:**
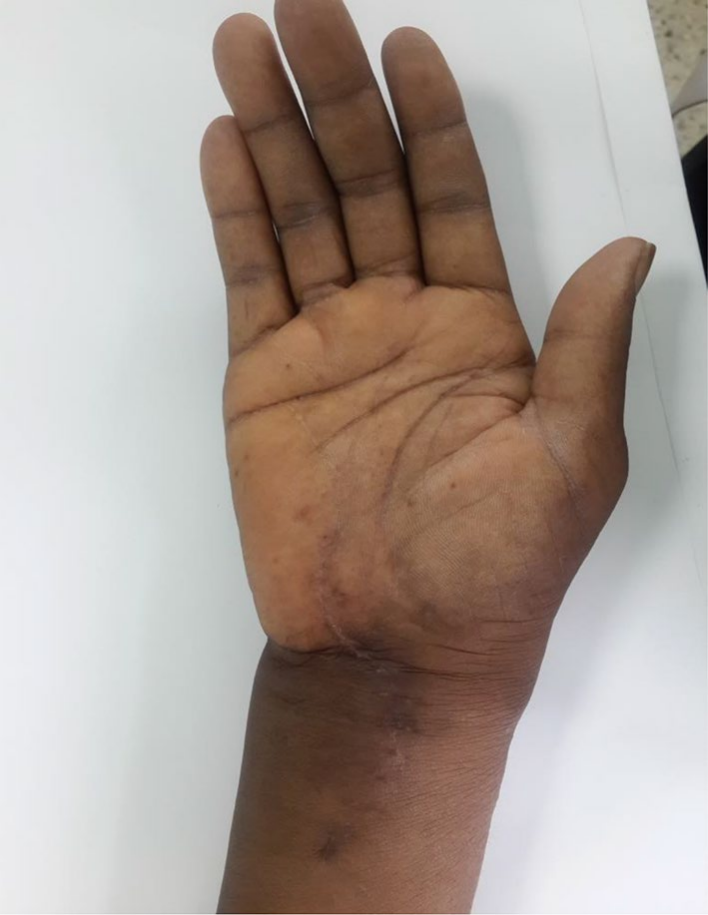
One year after surgery and there is no evidence of recurrence

**Fig. 7:**
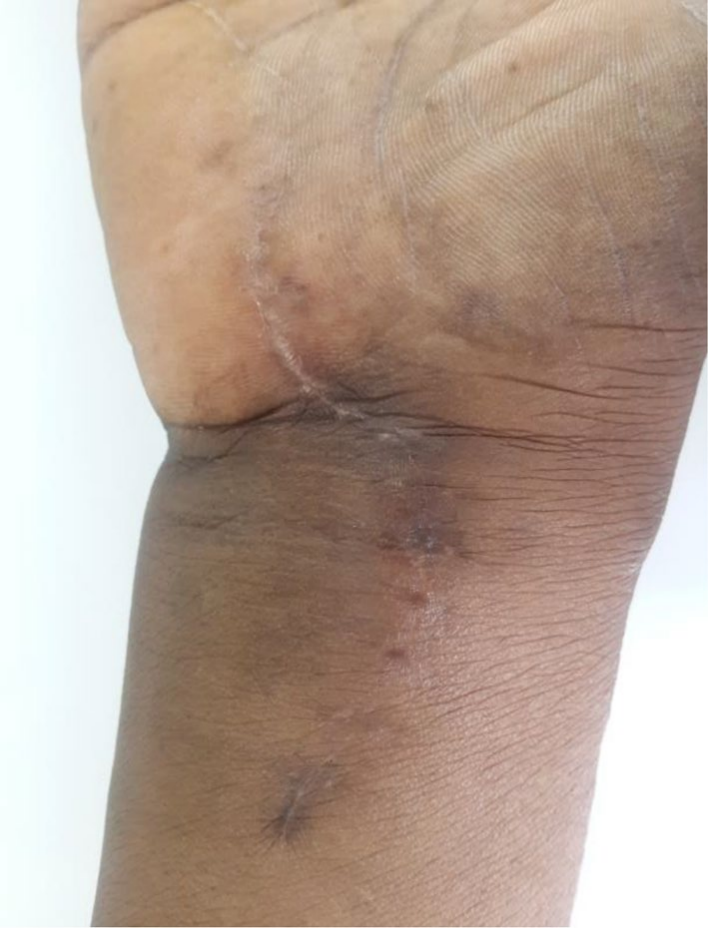
One year after surgery

Full occupational activities were allowed after 3 months when she has regained full strength in the hands. There was no recurrence at 1 year follow up (Figs. 6, 7).

## Discussion and conclusions

It was thought that, following the discovery of antituberculous medications; tuberculosis will not pose any danger to humans. However, it is still of public health importance due to the challenges with diagnosis and treatment [5]. It mainly affects the respiratory system but may affect other organs. About 14% are extra pulmonary [6].

Tuberculous tenosynovitis is an uncommon but well-documented condition [7]. It normally affects the wrist and volar aspect of the hand and accounts for 5% of cases of osteoarticular tuberculosis [8]. The mechanism of the infection may be through hematogenous spread from a primary site in the lungs, lymph nodes, genitourinary or bones or by direct inoculation [1]. Precipitating factors include trauma, overuse of the joint, old age, low socioeconomic status, malnutrition, and immunosuppression [9]. The mechanism in our patient was not clear but we believe it was due to immunosuppression probably as a result of diabetes, and overuse of the wrist, as she is a farmer. Males are commonly affected and the right hand and wrist are mainly involved [10]. Our patient was female though the handedness conforms to what is commonly reported in the literature.

Patients normally present with an insidious, slow growing, sausage-shaped mass along the involved tendon with or without pain [9]. Some may present with carpal tunnel syndrome (as in our patient) or with discharging sinus. Because the onset is gradual, most people present with a well-advanced disease.

The disease progresses through 3 histopathological stages depending on the duration, resistance of the person, and virulence of the infecting pathogens [9]. In the initial stages, there is the replacement of the tendon by granulation tissue. Subsequently, the sheath is obliterated by fibrous tissue. This is followed by the appearance of rice or melon bodies as a result of caseation. The tendons, at the end, may consist only of a few strands of tissue leading to spontaneous rupture [9]. Rice or melon bodies were first described by Reise in 1895 [11] and consist of fibrinous masses (tubercles) and are believed to be due to micro-infarction following inflammation and ischemia of the synovial sheath and are present in about 50% of cases [7, 12]. According to a study by Woon et al. [13], the presence of rice bodies together with millet or melon seed shaped lesions are diagnostic of tuberculous tenosynovitis. For this reason, one should be aware of the importance of loose bodies when excising harmless-looking wrist and palmar lesions [13].

Diagnosis of tuberculous tenosynovitis is usually delayed due to the numerous differential diagnosis including other atypical mycobacterial infections, tuberculosis, systemic lupus erythematosus (SLE), pyogenic infections, brucellosis, foreign body tenosynovitis, osteoarthritis and rheumatoid arthritis [7, 12, 14–17]. Ultimately, diagnosis is by open biopsy and culture of the histopathological specimen. This however takes time and may delay the diagnosis and treatment. Hence when a provisional diagnosis of tuberculous tenosynovitis is made, it is imperative to start anti-tuberculous treatment while awaiting the result [4]. The recommended management especially when the condition is associated with clinical evidence of carpal tunnel syndrome is surgical (that is excision biopsy involving thorough curettage, lavage, and synovectomy) [18, 19] to decompress the median nerve. Local recurrence is possible and about 50% of cases recur within 1 year of treatment [20].

In conclusion, tuberculous tenosynovitis of the wrist is uncommon. However, in developing countries like Ghana where tuberculosis is common, tuberculous tenosynovitis should be considered among the possible causes of chronic tenosynovitis around the wrist. This is to prevent delay in diagnosis and treatment with its attendant complications. Excision biopsy and anti-tuberculous chemotherapy should be considered without delay when it is associated with established clinical signs and symptoms of carpal tunnel syndrome.

## Abbreviations

HIV: human immunodeficiency virus
ESR: erythrocyte sedimentation rate
SLE: systemic lupus erythematosus
